# Signal transduction in astrocytes: Localization and release of inorganic polyphosphate

**DOI:** 10.1002/glia.23466

**Published:** 2018-09-07

**Authors:** Plamena R. Angelova, Kathrine Z. Iversen, Anja G. Teschemacher, Sergey Kasparov, Alexander V. Gourine, Andrey Y. Abramov

**Affiliations:** ^1^ Department of Molecular Neuroscience UCL Institute of Neurology, Queen Square London, WC1N 3BG United Kingdom; ^2^ Centre for Cardiovascular and Metabolic Neuroscience, Department of Neuroscience, Physiology, and Pharmacology University College London Gower Street, London, WC1E 6BT United Kingdom; ^3^ School of Physiology and Pharmacology University of Bristol, University Walk Bristol, BS8 1TD United Kingdom; ^4^ Baltic Federal University 2 Universitetskaya str, Kaliningrad, 236000 Russian Federation

**Keywords:** astrocytes, inorganic polyphosphate, lysosomes, mitochondria, VNUT

## Abstract

Inorganic polyphosphate (polyP) is present in every cell and is highly conserved from primeval times. In the mammalian cells, polyP plays multiple roles including control of cell bioenergetics and signal transduction. In the brain, polyP mediates signaling between astrocytes via activation of purinergic receptors, however, the mechanisms of polyP release remain unknown. Here we report identification of polyP‐containing vesicles in cortical astrocytes and the main triggers that evoke vesicular polyP release. In cultured astrocytes, polyP was localized predominantly within the intracellular vesicular compartments which express vesicular nucleotide transporter VNUT (putative ATP‐containing vesicles), but not within the compartments expressing vesicular glutamate transporter 2 (VGLUT2). The number of lysosomes which contain polyP was dependent on the conditions of astrocytes. Release of polyP from a proportion of lysosomes could be induced by calcium ionophores. In contrast, polyP release from the VNUT‐containing vesicles could be triggered by various physiological stimuli, such as pH changes, polyP induced polyP release and other stimuli which increase [Ca^2+^]_*i*_. These data suggest that astrocytes release polyP predominantly via exocytosis from the VNUT‐containing vesicles. © 2018 Wiley Periodicals, Inc.

## 1 INTRODUCTION

A number of complex signaling systems evolved to mediate communication between cells. Cell signaling is essential to maintain function of multicellular organisms in general, and brain information processing and control of homeostasis in particular. Signal transduction in the central nervous system is based on a signal from neuron to neuron or glial cell to glia through a well‐controlled system, which requires specific transmitter molecules. Astrocytes, the most numerous glial cells of the brain, respond to neurotransmitters and may contribute to synaptic information processing by releasing signaling molecules called “gliotransmitters” (Bazargani & Attwell, 2016).

ATP is a major signaling molecule released by astrocytes that enables communication between astrocytes and other brain cells, including neurones (Bowser & Khakh, 2007;Lalo, Rasooli‐Nejad, & Pankratov, 2014; Torres et al., 2012). Purinoceptors are expressed by astrocytes, and readily respond to the increases in extracellular ATP concentration (Angelova et al., 2015; Coco et al., 2003; Gourine et al., 2010). Regulated exocytosis of signaling molecules by astrocytes may involve different types of secretory organelles (synaptic‐like microvesicles, dense‐core vesicles, lysosomes, exosomes, and ectosomes (Oya et al., 2013; Verkhratsky, Matteoli, Parpura, Mothet, & Zorec, 2016).

Inorganic polyphosphate (polyP) is a polymeric molecule, consisting out of a number of orthophosphate residues and is present in all organisms. In prokaryotes and lower eukaryotes polyP have been found to plays multiple roles, including those similar to ATP (Angelova, Baev, Berezhnov, & Abramov, 2016; Holmstrom et al., 2013). In mammalian cells, polyP is involved in the mechanisms of cell death, blood coagulation, bone formation, mitochondrial metabolism, and calcium handling (Abramov et al., 2007; Angelova, Baev, et al., 2016; Morrissey, Choi, & Smith, 2012; Pavlov et al., 2010).

We have previously demonstrated that polyP might act as one of the gliotransmitters as the majority of astrocytes and a proportion (3%) of neurons respond to polyP with increases in cytosolic calcium. This effect is mediated through activation of P2Y_1_ receptors and stimulation of phospholipase C activity (Holmstrom et al., 2013). The concentration of polyP in the mammalian brain (50 µM) is much higher than the concentrations sufficient (10 nM–10 µM) to trigger Ca^2+^ signals in astrocytes (Kumble & Kornberg, 1995). This indicates that in the brain polyP is contained inside the cells, likely to be compartmentalized and released in a controlled manner. Previously polyP was found to be present in astroglial lysosomes and neuronal synaptosomes (Holmstrom et al., 2013; Stotz et al., 2014). However, in astrocytes only a small number of polyP containing lysosomes fused with the plasma membrane upon stimulation.

Recently we have developed novel highly specific probes to visualize polyP in living cells: JC‐D7 and JC‐D8, which track localization of polyP in living cells with high affinity (Angelova et al., 2014). In this study we used specific polyP indicators and novel molecular tools to determine localization of free polyP in specific cellular compartments of astrocytes and to study the mechanisms of polyP release in response to various stimuli.

## 2 MATERIALS AND METHODS

### 2.1 Cell culture

Primary cell cocultures of neurons and astrocytes were prepared as described in detail previously (Angelova, Ludtmann, et al., 2016; Turovsky et al., 2016) with modifications, from the midbrains and cerebral cortices of Sprague‐Dawley P3 rat pups or wildtype and LRRK2 knockout C57BL/6 mice (UCL breeding colony). Experimental procedures were performed in compliance with the United Kingdom Animals (Scientific Procedures) Act of 1986. After trypsinization of the tissue, the cells were plated on poly‐d‐lysine‐coated coverslips, according to the protocols described in (Deas et al., 2016) for 12 DIV. The cultures were transduced with either of the adenoviral vectors (AVV) AVV‐sGFAP‐mVNUT, AVV‐sGFAP‐VGLUT2‐EGFP, AVV‐sGFAP‐mKate‐CD63 or lentiviral vector (LVV) LVV‐EF1a‐TMPAP‐EGFP. Experiments were performed after 7–10 days of incubation with the virus.

### 2.2 Transduction

sGFAP is a transcriptionally enhanced, bidirectional, shortened glial fibrillary acidic protein promoter which drives transgene expression in astrocytes (Figueiredo et al., 2011; Gourine et al., 2010; Liu, Paton, & Kasparov, 2008). Fusions with EGFP or mKate2.7 allow detection of transgene expression via green or red fluorescence, respectively. The VGLUT2 (vesicular glutamate transporter; NM0534271; kind gift from P. Bezzi, Lausanne (Bezzi et al., 2004) is targeted to glutamatergic vesicles in astrocytes, while the mVNUT (vesicular nucleotide transporter; kind gift from T. Miyaji, Japan) delineates putative ATP‐containing vesicles (Sawada et al., 2008). CD63 is a lysosomal membrane protein which localizes to lysosome‐derived exocytotic vesicle‐like structures in astrocytes (CD63 clone BC063173 obtained from ImaGenes, Berlin (Metzelaar et al., 1991). LVV‐EF1a‐TMPAP‐EGFP drives expression of a fluorescently tagged plasma‐membrane‐anchored phosphatase (transmembrane prostatic acid phosphatase, TMPAP) which is designed to break down ATP within vesicles and extracellularly (Marina et al., 2013; Wells et al., 2015).

### 2.3 Fluorescent markers and live cell imaging

For identification of the colocalization of the vesicular/lysosomal compartments with polyP cell cultures, transduced to express VGLUT2–eGFP, VNUT–eGFP, TMPAP–eGFP, or CD63‐mKate were loaded with either DAPI (1 µM) or JC‐D7 (5 μM) or JC‐D8 (5 μM) for 30 min at room temperature in a HEPES‐buffered salt solution (HBSS) composed (mM): 156 NaCl, 3 KCl, 2 MgSO_4_, 1.25 KH_2_PO_4_, 2 CaCl_2_, 10 glucose, and 10 HEPES, pH 7.35.

Confocal images were obtained using Zeiss 710 CLSM microscope equipped with a META detection system and a 40 × oil immersion objective. JC‐D7/JC‐D8 fluorescence was determined with excitation at 405 nm and emission above 450 nm. For images in experiments comparing levels of fluorescence in different cells, the imaging setting were kept at the same level. The DAPI‐polyP (which was separated from DAPI–DNA/RNA) fluorescence was detected with excitation 405 nm and emission between 480 and 520 nm according to (Aschar‐Sobbi et al., 2008). Mitochondrial localization was identified using potential sensitive indicator tetramethylrhodamine (TMRM; Angelova et al., 2018). Cells were loaded for 40 min at room temperature and superfused with 20 nM TMRM, excited at 565 nm and imaged with a 580 nm emission filter as previously described. Measurements of fluorescence in astrocytes determined using different z‐tacks. Illumination intensity was kept to a minimum (at 0.1–0.2% of laser output) to avoid phototoxicity and the pinhole set to give an optical slice of ∼2 μm.

### 2.4 The images were analyzed using Zeiss ZEN software

To determine the percentage of lysosomes, mitochondria, VNUT or VGLUT vesicles that contain polyP, and the percentage of polyP localized within organelles or vesicles, colocalization analysis was performed on whole astrocytes. The analysis was based on the baseline of time‐scale experiments, to get the best representation. The Mander's overlap coefficients Mx and My were used for analysis, as Mx gives the percentage overlap of TMRM. Lysotracker Red of VNUT‐, CD63 or VGLUT with JC‐D8, and My gives the percentage overlap of JC‐D8 with organelles or vesicles, and disregards the absolute intensity (Dunn, Kamocka, & McDonald, 2011).

### 2.5 TIRF imaging

An Olympus total internal reflection fluorescence (TIRF) microscope was used to detect vesicular fusion events in astrocytes expressing VNUT–eGFP or CD63‐mKate, as described in detail previously (Angelova et al., 2015; Kasymov et al., 2013). Fluorescence was excited at 488 nm and collected at 500–530 nm for eGFP and excited at 488 nm and collected at 600–660 nm for mKate. The imaging setup was equipped with a high‐NA oil‐immersion objective (60×, 1.65 NA), an Olympus IX71 inverted microscope and a Hamamatsu CCD camera. Images were acquired using Olympus Cell$\hat{t}$ool software (Olympus) and later converted and analyzed with Zeiss Zen software (Zeiss).

### 2.6 Data analysis and statistics

Data and statistical analysis were performed using OriginPro (OriginLab, Northampton, USA) and GraphPad Prism (GraphPad Software, San Diego, USA) software. Data are presented as means expressed ± standard error of the mean (SEM).

## 3 RESULTS

### 3.1 Localization of polyP in astrocytes

#### 3.1.1 PolyP in mitochondria

**Figure 1:**
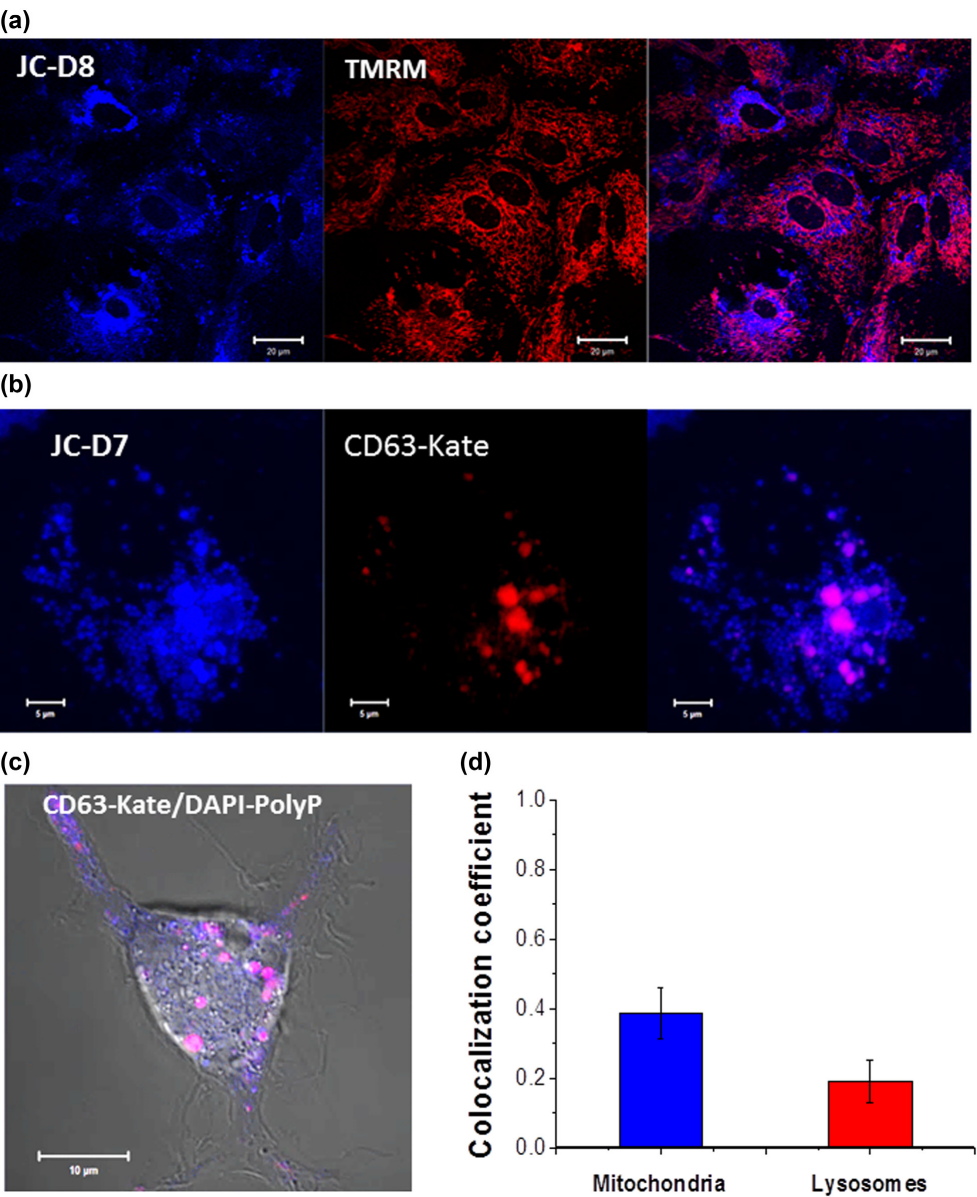
Localization of inorganic polyphosphate to mitochondria and lysosomes. (a) inorganic polyphosphate is found in mitochondria (polyP, JC‐D8, blue; mitochondria, TMRM, red). (b) Highly specific polyP signal (JC‐D7, blue) is colocalized with lysosomal (CD63‐mKate, red) signal. (c) DAPI‐polyP can also be used to track the localization of inorganic polyphosphate in lysosomes. d, Bar charts summarizing the quantification of colocalization (Mander's coefficient) of polyP signals with fluorescence signal out of mitochondria and lysosomes [Color figure can be viewed at http://wileyonlinelibrary.com]

PolyP in the mitochondria has its functional role; it plays an important role in energy metabolism and calcium handling (Baev, Negoda, & Abramov, 2017; Pavlov et al., 2010). In agreement with our previous report, we found that in cultured astrocytes the specific polyP indicator has higher intensity in the mitochondrial area (Angelova et al., 2014) (Figure 1a). Simultaneous measurement of mitochondrial (TMRM) and polyP (JC‐D8) signals showed that ∼40% of cellular polyP resides in mitochondria (here and below coefficient of colocalization is a Mander's X; 0.39 ± 0.08, *n* = 204 cells; Figure 1a,d). As we reported previously, the level of polyP in mitochondria is dependent on the energy state of mitochondria and can be modified within seconds (Pavlov et al., 2010).

#### 3.1.2 PolyP in lysosomes

We have previously reported that in cultured astrocytes DAPI‐polyP staining partially colocalizes with lysosomes labeled with LysoTracker Red (Holmstrom et al., 2013). We next labeled polyP with either DAPI‐polyP or JC‐D8 and genetically labeled lysosomes using a fusion of lysosomal LAMP3 protein with an adenoviral vector AVV‐sGFAP‐CD63‐mKate.

The number of labeled lysosomes and the localization of polyP in these organelles were found to be dependent on the cell state and condition. In cortical astrocytes under normal conditions the number of lysosomes containing polyP varied between ∼40% (0.42 ± 0.14, *n* = 54 cells for DAPI‐polyP/CD63‐mKate; Figure 1b) and ∼20% (0.19 ± 0.06; *n* = 62 cells; Figure 1c,d for JC‐D7 and CD63‐mKate).

**Figure 2:**
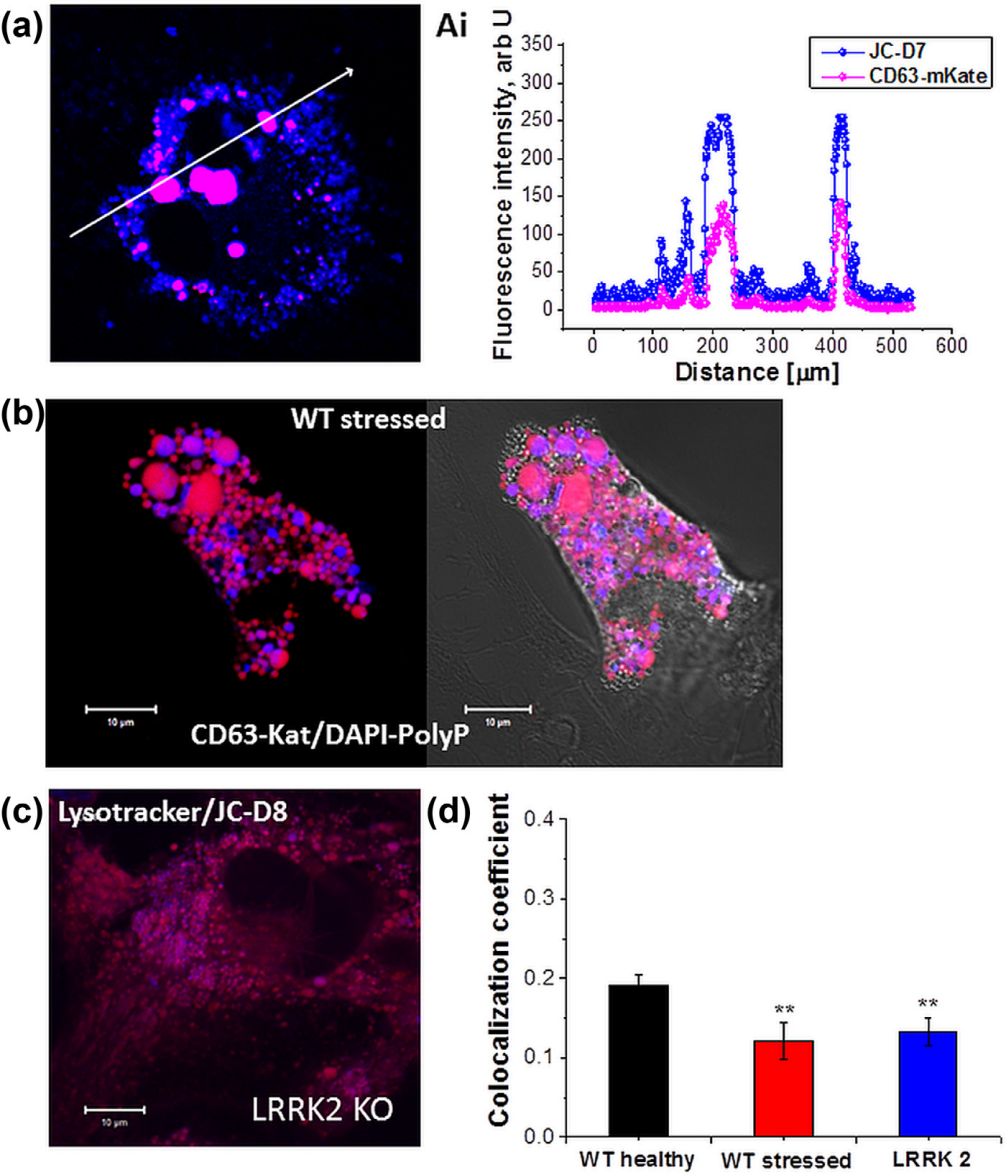
Localization of inorganic polyphosphate in healthy and stressed astrocytes. (a) Wildtype healthy astrocyte, labeled for lysosomal tetraspanin CD63 (LAMP‐3), fused with red fluorescent probe mKate and labeled for polyP with the JC‐D7 indicator. (Ai) colocalization profile of CD63‐mKate and JC‐D7. (b) Stressed wildtype astrocyte, expressing CD63‐mKate and labeled for polyP with DAPI‐polyP. Note the redistribution of the lysosomal signal. Colocalization of polyP (JC‐D8, blue) and lysosomes (LysoTracker Red, red) in cultured astrocytes from Parkinson's disease model LRRK2 knockout, known to have impaired lysosomal morphology and function, (c,d) histogram depicting the distribution of polyP in lysosomes from wildtype healthy (black bar), wildtype stressed (red bar) and LRRK2 knockout (blue bar) astrocytes. **p < .001 [Color figure can be viewed at http://wileyonlinelibrary.com]

We found that the number of labeled vesicles and the appearance of polyP in lysosomes were dependent on the health (Figure 2a,b1) and the age of the cells. Aged and starved (see Methods section) cortical astrocytes contained higher number of lysosomes with the same percentage of them showing colocalization of CD63‐mKate with polyP signal (Figure 2b,c).

Mutations in leucine‐rich repeat kinase 2 (LRRK2) are associated with a familial form of Parkinson's disease. This mutation manifests with defects in the autophagy/lysosomal degradation pathway (Hockey et al., 2015; Manzoni & Lewis, 2013). We found that in LRRK2 knockout mouse cortical astrocytes the percentage of polyP‐containing lysosomes was significantly decreased (Figure 2c,d; 0.19 ± 0.01, *n* = 51 for wildtype compared to 0.13 ± 0.02, *n* = 54 for LRRK2 knockout cultures; *p* < .001).

#### 3.1.3 PolyP in glutamate‐containing vesicles

**Figure 3:**
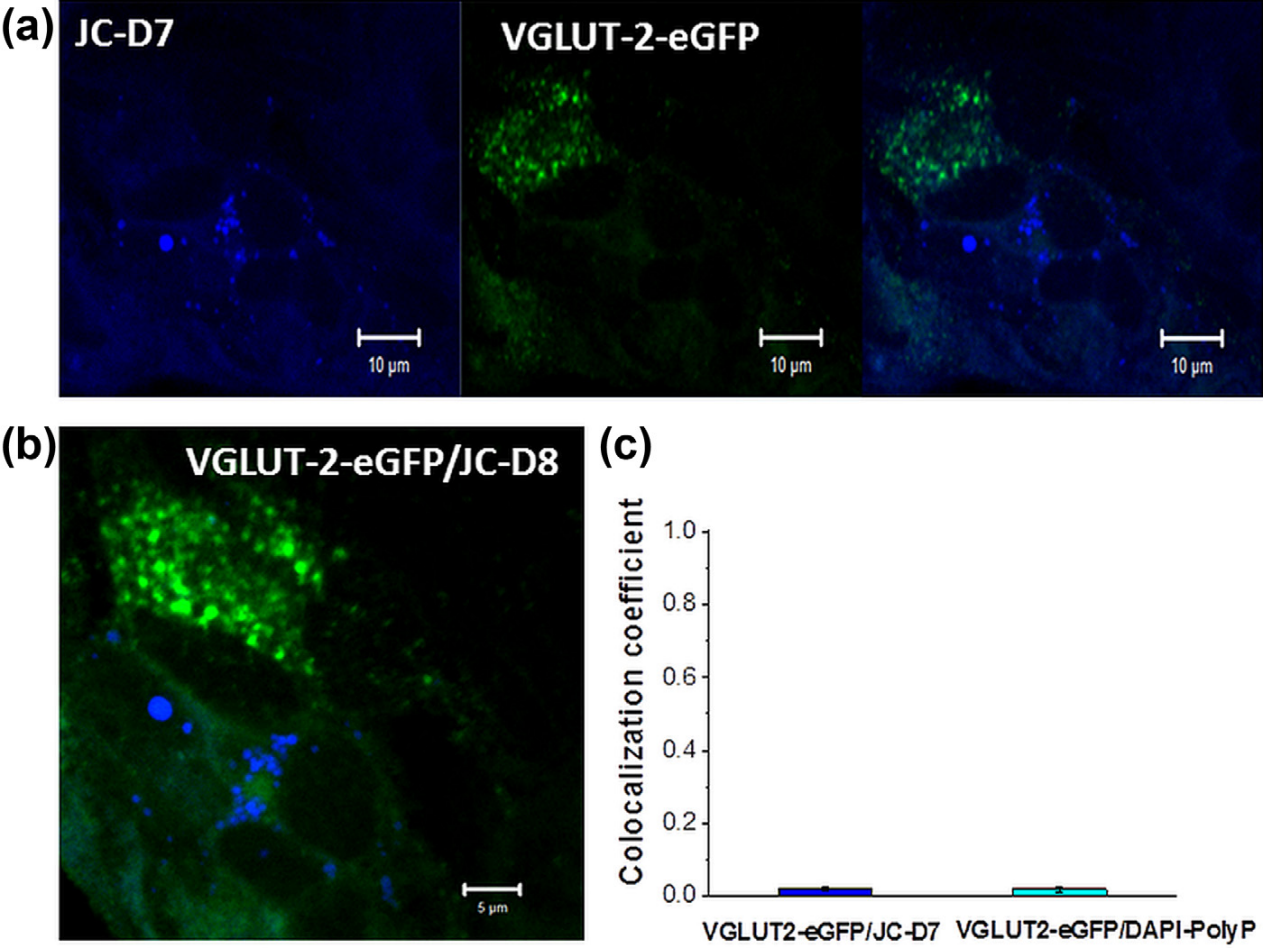
Lack of localization of polyP to glutamate‐containing vesicles (VGLUT2) in astrocytes from mixed primary culture. (a) Fluorescent image of an astrocyte, transduced to express eGFP–VGLUT2 and labeled with JC‐D8. (b) Colocalization image depicting the intensities of enhanced eGFP–VGLUT2 (green) and polyP indicator JC‐D7 (blue). Note lack of colocalization of the two signals. (c) Bar chart quantifying the colocalization coefficient (Mander's) of polyP and VGLUT signals [Color figure can be viewed at http://wileyonlinelibrary.com]

It is thought that astrocytes may release glutamate via exocytosis of VGLUT2‐containing vesicles. Transfection of cortical astrocytes with VGLUT2‐eGFP identified VGLUT2‐expressing vesicles (Figure 3a–c) but there was no colocalization of eGFP with either DAPI‐polyP or JC‐D7 (*n* = 113 and *n* = 143 cells, respectively; Figure 3a–d). These results indicate that astroglial vesicles which express VGLUT2 do not contain polyP.

#### 3.1.4 PolyP in ATP‐containing vesicles

**Figure 4:**
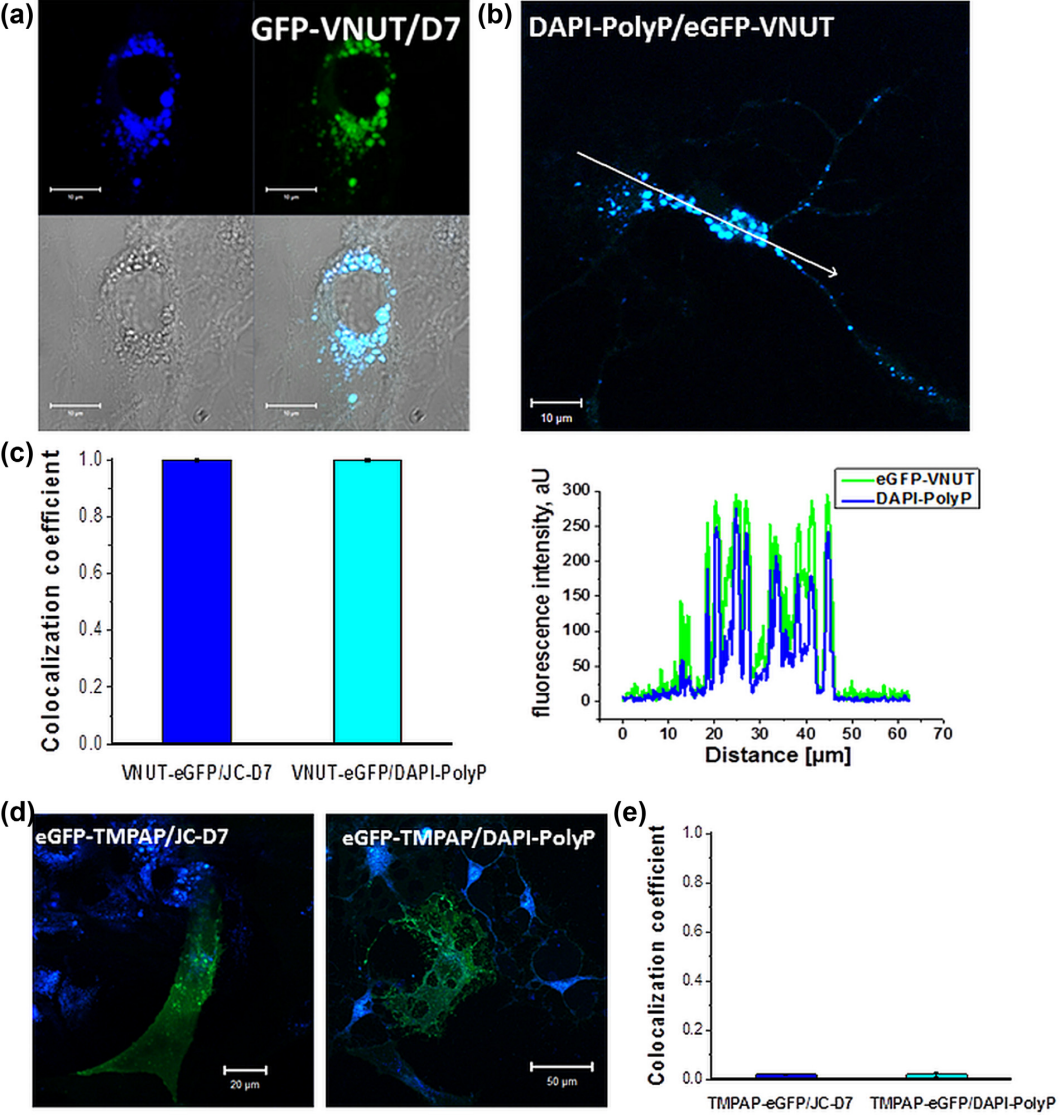
Localization of inorganic polyphosphate to ATP‐containing vesicles (expressing VNUT). (a,b) JC‐D8 and DAPI‐polyP fully colocalize with eGFP–VNUT signals in cultured astrocytes see summary in (c). (Bi) Colocalization profile of DAPI‐polyP and eGFP–VNUT. (d,e) JC‐D7 and DAPI‐polyP do not colocalize with the eGFP–TMPAP signal in rat astrocytes [Color figure can be viewed at http://wileyonlinelibrary.com]

PolyP may mediate signaling between astrocytes through activation of metabotropic P2Y_1_ receptors (Holmstrom et al., 2013). We next identified putative ATP‐containing vesicles, by transducing astrocytes to express eGFP‐tagged vesicular nucleotide transporter (VNUT) (eGFP–VNUT) and determined colocalization of eGFP fluorescence with the polyP marker JC‐D8 (Figure 4a). VNUT‐containing vesicles showed high degree of colocalization with JC‐D8‐polyP signal (0.9962 ± 0.0038, *n* = 141 cells; Figure 4a,c). DAPI‐polyP fluorescence also showed almost complete colocalization with VNUT–eGFP signal (0.9924 ± 0.0756, *n* = 151 cells; Figure 4 B, C) suggesting that in cultured astrocytes most of the VNUT‐containing vesicles contain polyP. Since eGFP fused with VGLUT2 show no colocalization with both polyP indicators (Figure 3), DAPI‐polyP or JC‐D8 signals are not contaminated by eGFP fluorescence.

JC‐D8 and DAPI specifically label free polyP and have no selectivity towards purines, including ATP or ADP (Angelova et al., 2014; Aschar‐Sobbi et al., 2008). To study the functional roles of ATP or ADP as signaling molecules, enzymatic depletion of ATP or ADP by phosphatases has been used in a number of studies (Wells et al., 2015). We have previously demonstrated that transmembrane prostatic acid phosphatase (TMPAP) prevents accumulation of ATP within the intracellular vesicular compartments (Wells et al., 2015). TMPAP should in theory also break down polyP and polyP degradation by TMPAP should be facilitated by the vesicular acidic environment. Astrocytes transduced to express TMPAP were found to be completely devoid of polyP not only within the vesicular compartments but also from mitochondria (Figure 4d,e).

### 3.2 Release stimuli for inorganic polyphosphate

#### 3.2.1 Release of polyP from lysosomes

**Figure 5:**
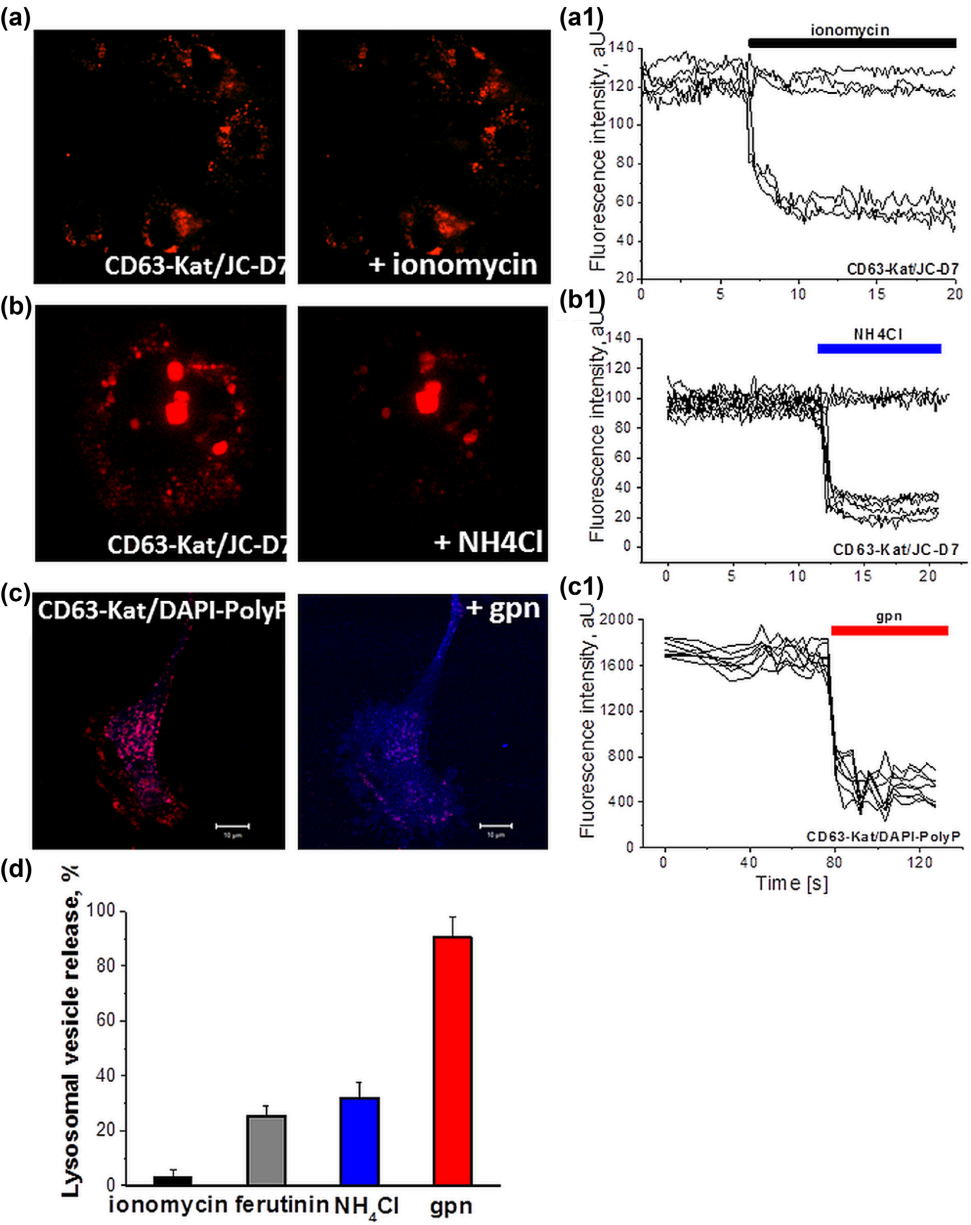
Total internal reflection microscopy (TIRF) imaging of lysosomal vesicle polyP release. Representative images (a) and traces (measurement of polyP in individual vesicles) (A1) depicting partial release of polyP from lysosomes upon application of calcium ionophore ionomycin (1 μM). Partial lysosomal polyP release upon acidification of the cytosol with NH4Cl (b,b1). (c,c1) Application of glycyl‐l‐phenylalanine‐β‐naphthylamide (GPN, 100 μM) results in the collapse of lysosomes and release of polyP to the cytoplasm (see increase of DAPI‐polyP fluorescence (blue) in cytosol). (d) Summary data showing release of polyP from the lysosomes upon different stimuli [Color figure can be viewed at http://wileyonlinelibrary.com]

We next recorded fusion of CD63‐expressing lysosomes in cultured cortical astrocytes using TIRF and confocal microscopy. To avoid changes in intensity of indicators due to the movement of vesicles inside the astrocytes but not because of release, in experiments with confocal microscopy, Z‐stacks with detection of polyP and vesicular indicators were performed. Previously we reported that application of the calcium ionophore ionomycin induces the release of polyP from polyP‐containing vesicles (Holmstrom et al., 2013). In agreement with our previous results, application of 5 µM ionomycin induced fusion of 3.5 ± 3.2% of CD63‐labeled lysosomes (*n* = 197 cells; Figure 5a,d). Considering the strong increase of [Ca^2+^]_*i*_ by ionomycin which can reflect rather pathological conditions, we next applied “mild” electrogenic calcium ionophore ferutinin (Abramov & Duchen, 2003; Abramov, Zamaraeva, Hagelgans, Azimov, & Krasilnikov, 2001; Zamaraeva et al., 1997). Ferutinin (30 µM) induced fusion of ∼25.3 ± 3.6% of CD63‐expressing intracellular compartments (*n* = 150 cells; Figure 5d). It should be noted that ferutinin can induce mitochondrial calcium overload and activate mitochondrial permeability transition pore, where polyP also play an important role (Abramov & Duchen, 2003; Abramov et al., 2007). Changes of intracellular pH induced by application of NH_4_Cl also triggered fusion of a significant proportion (32.1 ± 5.8%, *n* = 194 cells) of polyP‐containing lysosomes (Figure 5b,d). In order to further confirm lysosomal localization of polyP we next applied glycyl‐l‐phenylalanine‐β‐naphthylamide (GPN)—a substrate for lysosomal cathepsin C that can coordinately collapse the lysosomes. Application of GPN triggered release of polyP into the cytosol that was recorded as an increase of polyP‐JC‐D8 fluorescence (90.5 ± 7.5% *n* = 244 cells, Figure 5c,d). These data suggest that in astrocytes polyP is localized in lysosomes, but only a proportion of these compartments undergo exocytosis in response to the increases in intracellular Ca^2+^.

#### 3.2.2 Release of polyP from VNUT‐containing vesicles

**Figure 6:**
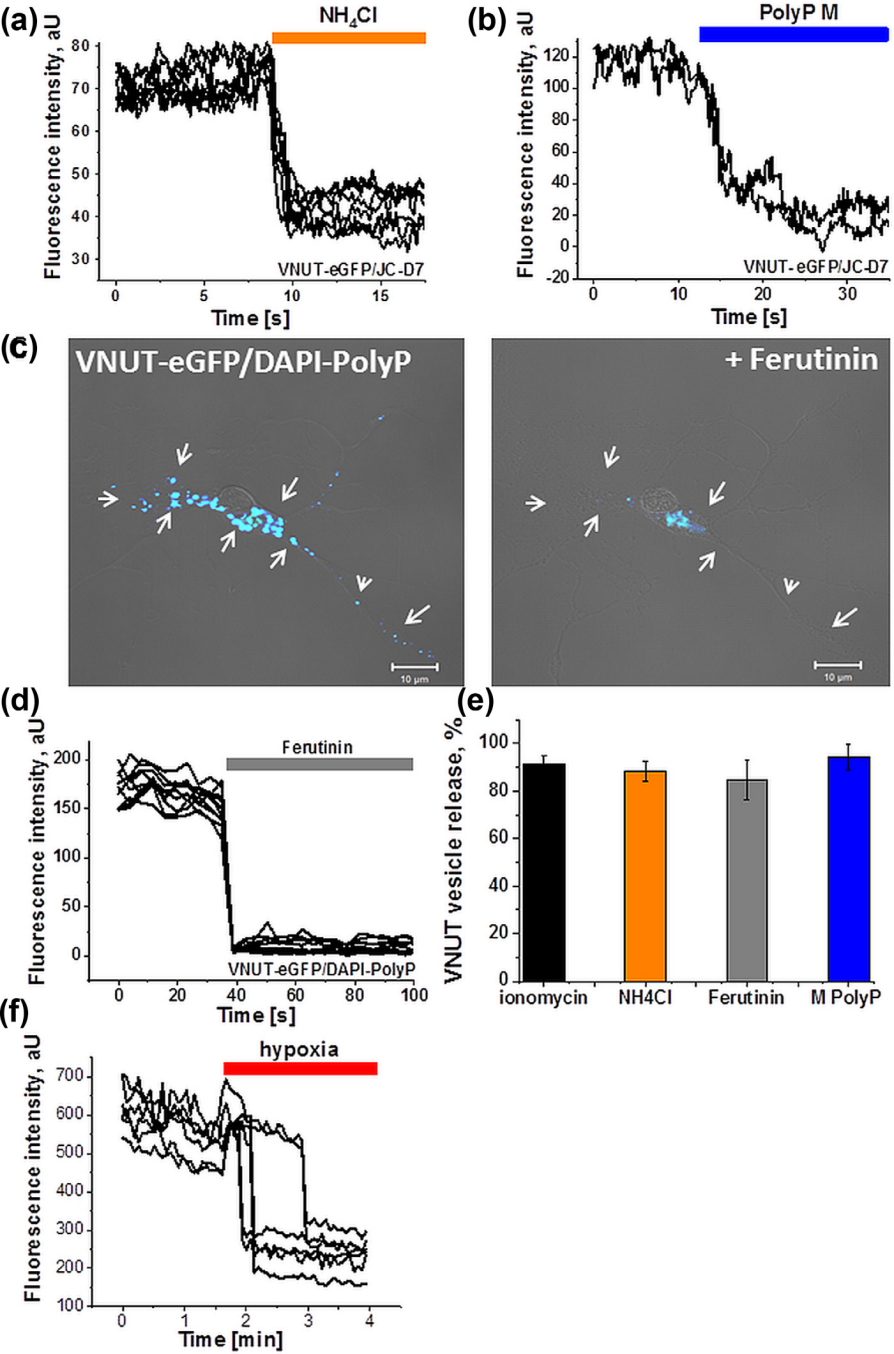
Release of polyphosphate from ATP‐containing (expressing VNUT) vesicles. TIRF microscopy reveals that various stimuli trigger fusion of VNUT‐expressing vesicles: (a) changes of the intracellular pH upon application of ammonium chloride (NH4Cl induced drop of polyP signal in vesicles; (b) exogenously applied medium‐chain polyphosphate (polyP M); (c,d) ferutinin, an electrogenic calcium ionophore (30 μM). (e) Quantification histogram depicting release of polyP from the VNUT‐containing vesicles upon different stimuli; (f) release of polyP from VNUT vesicles in response to short episode of hypoxia [Color figure can be viewed at http://wileyonlinelibrary.com]

We next determined whether polyP containing VNUT‐expressing vesicles undergo exocytosis in response to various stimuli. Fast pH changes induced by application of NH_4_Cl (alkalization) and washing it out (acidification) triggered fusion of 88.3 ± 3.9% of polyP‐containing VNUT vesicles (*n* = 163 cells, Figure 6a,e). Applications of the calcium ionophores ionomycin (1 µM; *n* = 251 cells) or ferutinin (30 µM; *n* = 215 cells) triggered fusion of almost the entire pool of polyP‐containing VNUT‐expressing vesicles (91.7 ± 3.4 and 84.8 ± 8.5%, respectively, Figure 6b,c,e).

Previously we reported that application of polyP to cortical astrocytes induces the release of polyP into the medium (Holmstrom et al., 2013). Here we found that polyP is released by exocytosis of the VNUT‐containing vesicles (Figure 6b,e) in response to application of medium length polyP (20 µM). Application of polyP led to a decrease in the intensity of the polyP‐JC‐D8 signal and triggered fusion of 94.3 ± 5.3% of VNUT‐containing vesicles (*n* = 209, Figure 6e). Thus, in astrocytes VNUT‐expressing vesicles represent the main pool of releasable polyP which participates in signal transduction.

Previously we found that even a short exposure of astrocytes to hypoxia results in an increase in intracellular calcium (Angelova et al., 2015). In the present study, a short episode of hypoxia induces the release of polyP by fusion of VNUT‐containing vesicles (*n* = 39, Figure 6f) suggesting that release of polyP from astrocytes may play a certain role in mediating the physiological response to brain hypoxia.

## 4 DISCUSSION

PolyP has been previously shown to be involved in a number of cellular processes (Angelova, Baev, et al., 2016; Morrissey et al., 2012; Schroder, Lorenz, Kurz, & Muller, 1999). Accumulation of polyP in cellular organelles and vesicles can indicate a specific signaling function. In this study, we found that ∼39% of the total pool of intracellular polyP in astrocytes is located in mitochondria (Figure 1) where it plays a role in bioenergetics (Angelova, Baev, et al., 2016; Pavlov et al., 2010) and calcium handling (Abramov et al., 2007; Baev et al., 2017; Elustondo et al., 2016). The level of polyP in mitochondria has been previously shown to be dependent on the metabolic state and the age of the cells (Pavlov et al., 2010). Astrocytes play a role of metabolic sensing and metabolic signaling (Marina et al., 2018) and the level of polyP may play important role in this process as signaling or/and metabolic molecule.

Although polyP is localized in 20‐40% of astroglial lysosomes (Figure 2), only a small percentage of these lysosomes undergo exocytosis in response to various stimuli. Lysosomes can elongate the polyP chain in human fibroblasts and granulocytes (Cowling & Birnboim, 1994; Manzoni & Lewis, 2013; Pisoni & Lindley, 1992). PolyP has been shown to play an important role in acidocalcisomes (Docampo & Moreno, 2011). Although the presence of these vesicles is in astrocytes is disputable, eukaryotic acidocalcisomes belong to the group of lysosome‐related organelles. They have a variety of functions, from the storage of cations and phosphorus to calcium signaling, autophagy, osmoregulation, blood coagulation, and inflammation (Docampo & Huang, 2016; Lander, Cordeiro, Huang, & Docampo, 2016)—all processes which can be regulating by lysosomal polyP in astrocytes.

The vesicular nucleotide transporter (VNUT) is a secretory vesicle protein which is responsible for the vesicular packaging of ATP (Sawada et al., 2008). Previously we and others reported that polyP could activate metabotropic P2Y_1_ nucleotide receptors (Dinarvand et al., 2014; Holmstrom et al., 2013). Here we detected not only fusion of the VNUT‐containing vesicles in response to various stimuli (Ca^2+^, pH, and activation of purinoceptors) but, importantly, release of polyP by exocytosis of these secretory vesicles. PolyP appears to play a role similar to that of ATP (as energy and signaling molecule), and here we found that in astrocytes these molecules could be potentially colocalized within the same intracellular vesicular compartments. At the moment we are not able to separate the functions which are specific to polyP and ATP but on the basis of indicator specificity and the effects induced by application of exogenous polyP can prove polyP induced polyP release—the signaling cascade which is typical for astrocytes. In summary, VNUT‐containing vesicles appear to be the prime source of the releasable pool of polyP.

Considering involvement of the interaction of astrocytes and neurons in neurodegenerative, neuropsychiatric, and other neurological disorders (Angelova & Abramov, 2014; Chiou, Lucassen, Sather, Kallianpur, & Connor, 2018; Liu, Teschemacher, & Kasparov, 2017), changes in polyP signaling may potentially be involved in the mechanism of pathology in CNS.

## CONFLICT OF INTEREST

The authors declare no conflict of interest.
